# Sex hormones and the risk of cardiovascular disease and mortality in male and female patients with chronic kidney disease: A systematic review and meta‐analysis

**DOI:** 10.14814/phy2.15490

**Published:** 2022-11-16

**Authors:** Ester S. Oh, Cortney N. Steele, Zhiying You, Kristen L. Nowak, Anna J. Jovanovich

**Affiliations:** ^1^ Division of Renal Diseases and Hypertension University of Colorado Anschutz Medical Campus Aurora Colorado USA; ^2^ VA Eastern Colorado Healthcare System Aurora Colorado USA

**Keywords:** cardiovascular disease, chronic kidney disease, mortality, sex hormone, systematic review

## Abstract

Patients with chronic kidney disease (CKD) commonly experience sex hormone disturbances, which may be associated with the risk of cardiovascular disease (CVD) and mortality. This review aimed to systematically evaluate current findings on the association of sex hormone levels with the risk of CVD events and mortality (CVD and all‐cause) in the CKD population. Articles were systematically searched in CINAHL, Cochrane, and PubMed. A total of 1739 articles were independently screened by two reviewers and 17 prospective cohort studies were included. The clinical conditions of the patients were those with non‐dialysis CKD [mean/median estimated glomerular filtration rate (eGFR) between 15–51 ml/min/1.73 m^2^] and those on chronic dialysis (mean/median vintage between 6–125 months). The sample size ranged from 111 to 2419 and the mean/median age of subjects ranged from 52 to 72 years. The sex hormones studied were testosterone, estradiol, prolactin, dehydroepiandrosterone sulfate, and relaxin. A random‐effects model was used to generate a pooled hazard ratio (HR) to evaluate the association of total testosterone levels with the risk of CVD and all‐cause mortality. Most studies examined total testosterone levels (11 out of 17 studies) and studied only male patients (12 out of 17 studies). A lower total testosterone level was associated with a higher risk of CVD mortality [HR 4.37 (95% CI 1.40–13.65)] and all‐cause mortality [1.96 (1.35–2.83)] in males with CKD. To conclude, there is a strong need for additional studies examining the association of sex hormones with cardiovascular and mortality risk in female patients with CKD.

## Graphical abstract

Patients with chronic kidney disease commonly experience sex hormone disturbances, which may be associated with the risk of cardiovascular disease and mortality. In the current systematic review, we found most studies examined total testosterone levels (11 out of 17 studies) and studied only male patients (12 out of 17 studies). A lower total testosterone level was associated with a higher risk of CVD and all‐cause mortality in males with CKD. There is a strong need for additional studies examining the association of sex hormones with cardiovascular and mortality risk in female patients with CKD.

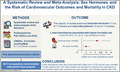

## 1 INTRODUCTION

Patients with chronic kidney disease (CKD) commonly experience sexual and gonadal dysfunction (Rathi & Ramachandran, 2012). The reproductive system is regulated by the hypothalamic–pituitary‐gonadal (ovarian/testicular) axis, and kidney disease adversely influences this axis at multiple levels (Rathi & Ramachandran, 2012). Both in male and female patients with CKD, sex hormone disturbances appear to be in part a consequence of defects in the hypothalamus, leading to a loss of cyclic release of gonadotropin‐releasing hormone (GnRH), resulting in elevated gonadotropin levels [luteinizing hormone (LH) and follicle‐stimulating hormone (FSH)] and reduced endogenous production of primary sex hormones, particularly testosterone and estradiol (Rathi & Ramachandran, 2012). Testosterone levels decrease in parallel with the reduction of estimated glomerular filtration rate (eGFR) in male patients with non‐dialysis CKD (Yilmaz et al., 2011) and testosterone deficiency (defined as total testosterone <10 nmol/L) is commonly observed in male patients on hemodialysis (Carrero et al., 2011). In younger female patients with CKD, sex hormone disturbances induced by CKD often lead to amenorrhea and menstrual irregularities (Vellanki & Kramer, 2019).

Endogenous sex hormone levels, especially testosterone and estradiol, are known to be associated with the risk of cardiovascular disease (CVD) and mortality in both males and females without CKD (Kaur & Werstuck, 2021; Zhao et al., 2018). Reduced endogenous production of sex hormones with CKD as well as aging contribute to increased risk of CVD and mortality. Several studies demonstrated association of endogenous total testosterone and estradiol concentrations with the risk of cardiovascular outcomes and mortality in male and female patients with CKD (Bello et al., 2014; Carrero et al., 2009, 2011; Grossmann et al., 2015; Gungor et al., 2010; Khurana et al., 2014; Kyriazis et al., 2011; Nakashima et al., 2017; Wu et al., 2018; Yilmaz et al., 2011; Yu et al., 2017). Most, but not all, studies reported an inverse association of testosterone and estradiol levels with the risk of CVD and all‐cause mortality. Systematically evaluating the current evidence and pooling the data from these studies may clarify the conflicting results and provide an insight into the literature gap, as well as identify types of sex hormones examined and subject characteristics of included participants (e.g., age, sex, dialysis or non‐dialysis, dialysis modality, and vintage).

Accordingly, the primary goal of this systematic review and meta‐analysis was to systematically evaluate current findings from studies that examined the association of circulating endogenous sex hormone levels, including but not limited to testosterone and estradiol, with the risk of CVD events, CVD mortality, and all‐cause mortality in male and female patients with CKD.

## 2 METHODS

### 2.1 Protocol registration

The current systematic review was guided by the standards of the Preferred Reporting Items for Systematic Reviews and Meta‐Analysis (PRISMA) 2020 statement (Page et al., 2021) Table S1. This study was registered in PROSPERO (CRD42021287427) prior to screening and selecting articles.

### 2.2 Search strategy

The search strategy included terms to reflect the key concepts of CKD (population), sex hormones (exposure), and CVD events, CVD mortality and all‐cause mortality (outcome) in humans. Data search terms, filters and number of results are provided in Table S2. The search was conducted using three databases including Cumulative Index for Nursing and Allied Health (CINAHL), Cochrane Central Register of Controlled Trials, and PubMed, and article records were managed in EndNote 20 (Clarivate Analytics).

### 2.3 Inclusion and exclusion criteria

Before the study selection, inclusion and exclusion criteria were created (Table 1). The eligible population was adults ≥18 years and individuals with impaired kidney function. Eligible studies were cohort studies that reported hazard ratio (HR), odds ratio (OR), or relative risk (RR) of the association between circulating sex hormone levels and the risk of CVD events, CVD mortality, and all‐cause mortality. Animal studies, reviews, conference abstracts, editorials, commentaries, and book chapters were excluded.

**TABLE 1:** Inclusion and exclusion criteria

Components	Inclusion criteria	Exclusion criteria
Date range	2/1/1955–6/2/2022	—
Language range	English only	—
Population	Adults (≥18 years old) Individuals with impaired kidney function (proteinuria, albuminuria, CKD, and dialysis)	Kidney transplant recipients
Intervention	Circulating sex hormone levels	—
Control	—	—
Outcome	HR, OR, or RR of CVD events, CVD mortality, and all‐cause mortality	—
Study design	Cohort studies	Animal studies
Publication format	—	Reviews Conference abstracts Editorials Commentaries Book chapters

Abbreviations: CKD, chronic kidney disease; CVD, cardiovascular disease; HR, hazard ratio; OR, odds ratio; RR, relative risk.

### 2.4 Study selection and data extraction

Study selection and data extract process has been conducted as previously described (Oh et al., 2021). ESO and CNS independently screened articles by titles and abstracts based on the inclusion and exclusion criteria and identified the search in duplicate. Inter‐rater reliability was calculated using Cohen's kappa method (Park & Kim, 2015). Any discrepancies were resolved by AJJ and KLN.

Data were extracted by ESO into a standardized spreadsheet and verified by CNS. Extracted data included first author, publication year, country of the cohort study conducted, study design, clinical condition and complications of the study population, number of participants, sex and mean/median age, race and ethnicity, mean/median eGFR, and vintage (if applicable). For exposure variables, the type of sex hormones and the measurement scale of the sex hormone as an exposure variable (e.g., continuous vs. categorical) were extracted. For outcome variables, HR, OR, or RR (95% confidence interval [CI]) of CVD events, CVD mortality, or all‐cause mortality were extracted. For studies that had multiple measurement scales of the exposure and/or outcome variables, the data were separately extracted.

### 2.5 Quality assessment

The NIH National Heart, Lung, and Blood Institute (NHLBI), 2013 Quality Assessment Tool for Observational Cohort and Cross‐sectional Studies was used to assess the quality of the included studies. The quality was rated as excellent (12–14 points), good (8–11 points), fair (4–7 points), and poor (0–3 points). ESO and CNS independently assessed the quality of included studies in duplicate. Any discrepancies were resolved by discussion.

### 2.6 Statistical analysis

As previously described (Araujo et al., 2011), HRs were converted to a uniform scale since the predictors (testosterone level) were originally presented with different cut‐off levels (continuous, binary, tertiles, quartiles, or quintiles). The scaling method assumes that total testosterone level is normally distributed and its association with HR is log‐linear, which was verified in previous study (Araujo et al., 2007). In brief, for studies that reported HRs of continuously modeled testosterone levels, we used standard deviation (SD) of the log value. Two studies were not able to be included in the meta‐analysis because the SD was not reported (Grossmann et al., 2015; Yilmaz et al., 2011). For studies that reported HRs of equal binary, tertile, quartile, and quintile cut‐off of testosterone levels, we used a scaling factor of 1.37 (2.18/1.59), 1.00 (2.18/2.18), 0.86 (2.18/2.54), and 0.78 (2.18/2.80), respectively. For studies that reported HR of unequal cut‐off of testosterone level, we used study‐specific scaling factors, calculated as 2.18/𝑥, where 𝑥 is the difference in means between the unequal testosterone cut‐off levels. These uniform scaled HRs and 95% CIs were pooled to evaluate the association between testosterone levels and the risk of CVD and all‐cause mortality using Review Manager (RevMan), 2014 version 5.4.1 and NCSS 2022, version 22.0.3. HRs of the association between testosterone levels and the risk of CVD events from two studies were unable to be pooled because the SD of the log value was not available from one study (Yilmaz et al., 2011). A random effects model was used to generate a pooled effect of estimates allowing for differences in observed outcomes (risk of CVD and all‐cause mortality) across studies. Statistical heterogeneity was evaluated using the among‐study variance (tau‐squared, 𝜏^2^), chi‐squared (χ^2^) test, and Higgin's *I* ^2^ statistics. Funnel plots were generated using RevMan version 5.4.1 to examine publication bias of studies included in the meta‐analysis. Statistical significance was accepted at *p* < 0.05.

## 3 RESULTS

### 3.1 Study selection

**FIGURE 1:**
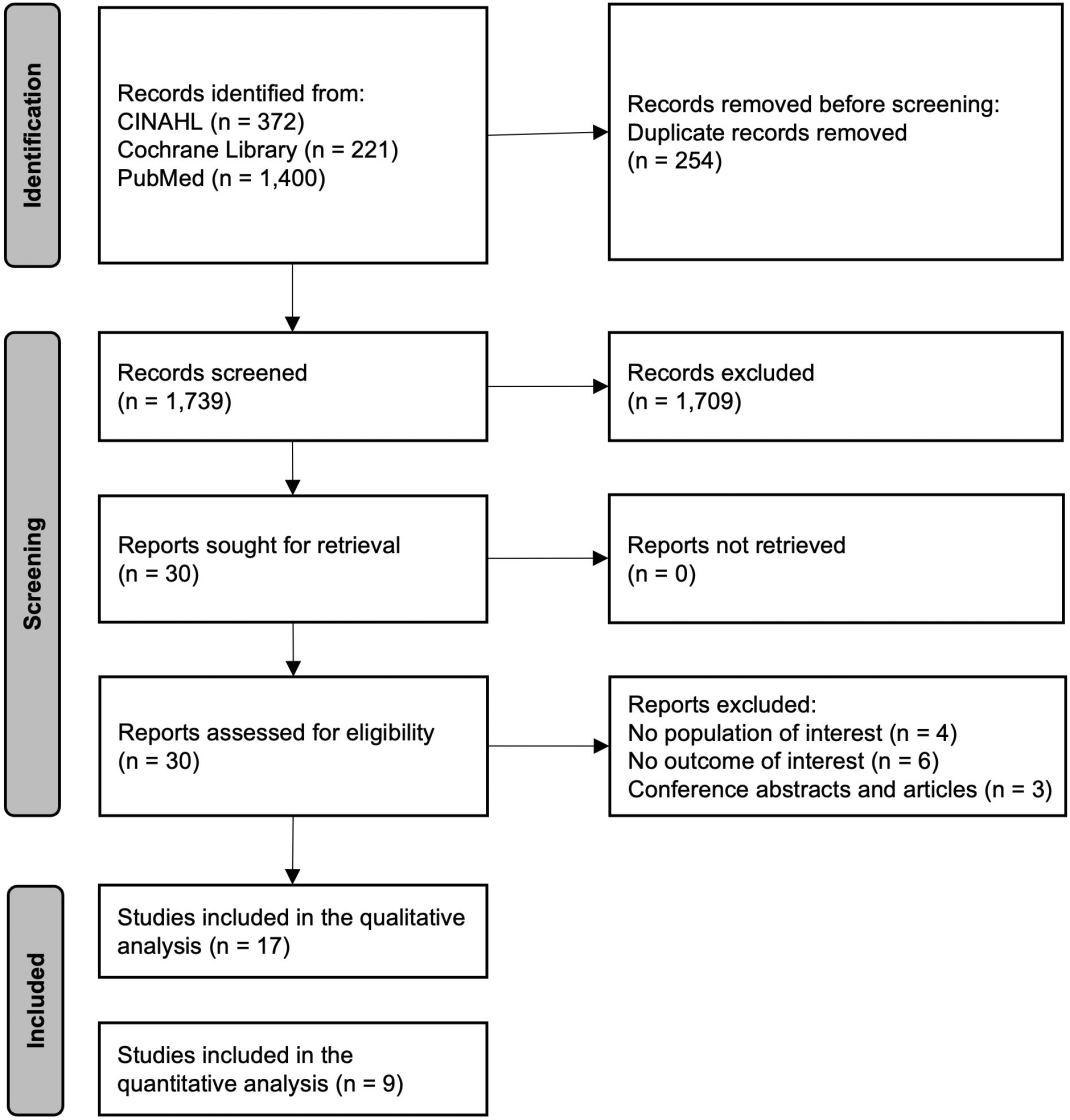
Preferred reporting items for systematic reviews and meta‐analyses (PRISMA) flow diagram of included studies.

We identified 1993 articles from database searches, leaving a total of 1739 articles for title/abstract screening after de‐duplication. We excluded 1709 articles according to the inclusion and exclusion criteria (Table 1), leaving 30 articles for full‐text review. We additionally removed 13 articles because they did not meet the eligibility criteria [no population of interest (*n* = 4), no outcome of interest (*n* = 6), and conference abstracts and review articles (*n* = 3)] (Figure 1). Thus, a total of 17 studies were included in this systematic review and meta‐analysis. There was a high inter‐rater reliability (κ = 0.71 for title/abstract screening and κ = 0.93 for fully text selection).

### 3.2 Characteristics of included studies

The current review included 17 prospective cohort studies that examined the association between circulating sex hormone concentrations and the risk of CVD events, CVD mortality, and all‐cause mortality in patients with non‐dialysis and dialysis CKD (Table 2). The clinical conditions of the patients included those with non‐dialysis CKD (mean/median eGFR between 15–51 ml/min/1.73 m^2^) and those on chronic dialysis (mean/median vintage between 6–125 months). The commonly reported comorbidities were diabetes (ranged between 17–62%) and CVD (ranged between 16–66%). The types of sex hormones included total and free testosterone (*n* = 11), estradiol (*n* = 2), prolactin (*n* = 1), dehydroepiandrosterone sulfate (DHEA‐S) (*n* = 2), and relaxin (*n* = 1). The sample size ranged from 111 to 2419 and the mean/median age from 52 to 72 years. The mean/median follow‐up period ranged from 20 to 102 months. Only 8 of 17 studies included female patients to evaluate the association between sex hormone levels and the risk of CVD events and mortality. Of the 8 studies that included females, only 5 reported outcomes of the female patients.

**TABLE 2:** Characteristics of included studies on the association between sex hormone and the risk of CVD events, CVD mortality, and all‐cause mortality

Author, year, country	Study design	Subject characteristics						Exposure	Outcomes	
		Clinical condition	Comorbidity, %	*N* (male %)	Age, years^a^	eGFR, ml/min/1.73 m^2a^	Vintage, months^a^	Exposure details	Outcome details	Adjustment
Testosterone										
Bello et al. (2014), Canada	Prospective cohort	Hemodialysis	Cancer, 17% CHF, 19% MI, 25 DM, 50% Stroke, 10% Dementia, 2% Liver disease, 7% COPD, 13% PVD, 13% HIV, 0.3%	623 (100%)	61	10	initiating	*TT (tertile)* T1: <231 ng/dL T2: 231–346 ng/dL T3: >346 ng/dL (ref.)	OR (95% CI); F/U = 20 months Incident CVD events, *N* = 98 (20%) HR (95% CI); F/U = 20 months All‐cause mortality, *N* = 166 (27%)	Age, race, smoking status, BMI, SHBG, cancer, and DM
Carrero et al. (2009), Sweden	Prospective cohort	Hemodialysis	DM, 27% CVD, 66%	126 (100%)	63	NA	27	*TT (binary)* T1: <8.1 nmol/L (ref) T2: 8.1–12.0 nmol/L T3: >12.0 nmol/L	HR (95% CI); F/U = 41 months CVD mortality, *N* = 38 (30%) All‐cause mortality, *N* = 65 (52%)	Age, SHBG, baseline CVD, DM, ACEI/ARB medication, IL‐6, albumin, and creatinine
Carrero et al.,(2011), Sweden	Prospective cohort	Hemodialysis	DM, 34% CVD, 57%	260 (100%)	59	NA	13	*TT (binary)* B1: <10 nmol/L B2: ≥10 nmol/L (ref.)	OR (95% CI); F/U = 36 months CVD events, N = ND All‐cause mortality, *N* = 88 (34%)	‐
Grossmann et al. (2015), Australia^b^	Prospective cohort	Non‐dialysis stage 3–4 CKD, undergoing dialysis, and kidney transplant recipients	DM, 22% CVD, 28% HTN, 90%	221 (65%)	59	15	28	*TT (per 1 nmol/L)*	HR (95% CI); F/U = 102 months All‐cause mortality, *N* = 87 (39%) Male all‐cause mortality, *N* = 52 Female all‐cause mortality, *N* = 35	Age, DM, pre‐existing CVD, renal disease status, BMI, CRP, and albumin
Gungor et al. (2010), Turkey	Prospective cohort	Hemodialysis	DM, 23% CVD, 16%	420 (100%)	53	NA	54	*TT (tertile)* T1: <6.8 nmol/L T2: 6.8–10.1 nmol/L T3: >10.1 nmol/L (ref.) *TT (per 1 nmol/L)*	HR (95% CI); F/U = 32 months All‐cause mortality, *N* = 104 (25%)	Age, vintage, diabetes, CVD, BMI, albumin, creatinine, and CRP
Khurana et al. (2014), US	Prospective cohort	Non‐dialysis stage 3–4 CKD	DM, 31% HTN, 93% Cerebrovascular disease, 10% Cancer, 28% CAD, 30% CHF, 14% Hyperlipidemia, 85%	2419 (100%)	67	49	ND	*TT (binary)* B1: <350 ng/dL or TRT B2: ≥350 ng/dL (ref.) *TT (quintile)* Qi1: 100–226 ng/dL Qi2: 227–305 ng/dL Qi3: 306–392 ng/dL Qi4: 393–511 ng/dL Qi5: 512–3153 ng/dL (ref.) *TT (per 1 log unit)*	HR (95% CI); F/U = 28 months All‐cause mortality, *N* = 357 (15%)	Age, race, eGFR, DM, HTN, cerebrovascular disease, CAD, CHF, hyperlipidemia, malignancy, BMI category, smoking status, albumin, and testosterone medication
Kyriazis et al. (2011), Greece	Prospective cohort	Hemodialysis	DM, 17% CVD, 57% HTN, 50%	111 (100%)	65	NA	42	*TT (binary)* B1: <8 nmol/L B2: ≥8 nmol/L (ref.) *TT (tertile)* T1: <5.2 nmol/L T2 + T3: ≥5.2 nmol/L (ref.) *FT (tertile)* T1: <0.21 nmol/L T2 + T3: ≥0.21 nmol/L (ref.)	HR (95% CI); F/U = 37 months CVD mortality, *N* = 28 (25%) All‐cause mortality, *N* = 49 (44%)	Age, BMI, baseline CVD history, vintage, serum albumin, CRP, and PWV
Nakashima et al. (2017), Japan	Prospective cohort	Hemodialysis	DM, 41% CVD, 20%	902 (100%)	63	NA	81	*TT (tertile)* T1: <9.05 nmol/L T2: 9.05–13.7 nmol/L T3: >13.7 nmol/L (ref.)	HR (95% CI); F/U = 25 months CVD events, *N* = 151 (17%) All‐cause mortality, *N* = 123 (14%)	Age, BMI, albumin, creatinine, CRP, SHBG, ACEI/ARB medication, DM, and history of CVD
Wu et al. (2018), Taiwan	Prospective cohort	Hemodialysis	DM, 38% HTN, 65% CHF, 10% Cancer, 7% CAD, 19%	137 (100%)	72	NA	23	*TT (tertile)* T1: <6.25 nmol/L T2 + T3: ≥6.25 nmol/L (ref.)	HR (95% CI); F/U = 23 months CVD mortality, *N* = 36 (26%) All‐cause mortality, *N* = 61 (45%)	DM, HTN, CRP, albumin, skeletal muscle mass index, creatinine, hemoglobin, testosterone, and body composition
Yilmaz et al. (2011), Turkey	Prospective cohort	Non‐dialysis CKD (stage 1–5)	DM, 22% CVD, 33%	239 (100%)	52	51	ND	*TT (per 10 nmol/L) FT (per 10 pg/mL)*	HR (95% CI); F/U = 31 months CVD events, *N* = 72 (30%)	Age, eGFR, DM, previous CVD, CRP, albumin, and FMD
Yu et al. (2017), US	Prospective cohort	Hemodialysis and peritoneal dialysis	DM, 62% CHF, 47% CHD, 19%	624 (100%)	58	NA	12	*TT (quartile)* Q1: 0–190 mg/dL Q2: 191–296 mg/dL Q3: 297–423 mg/dL (ref.) Q4: 425–1644 mg/dL	HR (95% CI); F/U = 90 months All‐cause mortality, *N* = 108 (17%)	Calendar quarter of study entry, age, sex, race/ethnicity, and DM, vintage, cause of ESKD, modality, dialysis access, CHF, CHD, and albumin
Estradiol										
Ramesh et al. (2020), Canada	Prospective cohort	Hemodialysis	DM, 53% HTN, 87% CAD, 28%	476 (0%)	60	NA	Initiating	*E (quintile)* Q1: <24 pmol/L (ref.) Q2: 24–47 pmol/L Q3: 48–82 pmol/L) Q4: 83–157 pmol/L Q5: >157 pmol/L	HR (95% CI); F/U = 35 months CVD mortality, *N* = 73 (15%) All‐cause mortality, *N* = 237 (50%)	Age, BMI, DM, smoking status, HTN, history of CAD, prolactin, and glomerulonephritis
Tanrisev et al. (2013), Turkey	Prospective cohort	Hemodialysis	DM, 49% CVD, 21%	147 (0%)	64	NA	35	*E (tertile)* T1: <21 pg/mL T2: 21–30 pg/mL (ref.) T3: >30 pg/mL	HR (95% CI); F/U = 32 months CVD mortality, *N* = 22 (15%) All‐cause mortality, *N* = 52 (35%)	Age, DM, BMI, urea reduction rate, and hs‐CRP
Prolactin										
Carrero et al. (2012), Greece	Prospective cohort	Non‐dialysis CKD	DM, 23% CVD, 50%	457 (50%)	52	ND	ND	*Prolactin (per 10 ng/mL)*	HR (95% CI); F/U = 38 months CVD events, *N* = 146 (32%) CVD mortality, *N* = 40 (9%) All‐cause mortality, *N* = 45 (10%)	Age, sex, smoking status, eGFR, M, CVD, MAP, CRP, albumin, FMD, IMT
Carrero et al. (2012), Turkey	Prospective cohort	Hemodialysis	DM, 18% CVD, 49%	173 (64%)	65	NA	≥6	*Prolactin (per 10 ng/mL)*	HR (95% CI); F/U = 49 months CVD mortality, *N* = 47 (27%) All‐cause mortality, *N* = 79 (46%)	Age, sex, smoking status, eGFR, CM, CVD, MAP, CRP, albumin, FMD, IMT
Dehydroepiandrosterone sulfate (DHEA‐S)										
Hsu et al. (2012), Taiwan^b^	Prospective cohort	Hemodialysis	DM, 37% CAD, 19% CHF, 22% PAD, 13% COPD, 9% Peptic ulcer disease, 35% Stroke, 10% Cancer, 9%	200 (47%)	59	NA	85	*DHEA‐S (binary)* B1: <790 ng/mL B2: ≥790 ng/mL (ref.) *DHEA‐S (continuous)*	HR (95% CI); F/U = 38 months CVD mortality, *N* = 27 (14%) Male CVD mortality, *N* = 11 Female CVD mortality, *N* = 16 All‐cause mortality, *N* = 60 (30%) Male all‐cause mortality, *N* = 35 Female all‐cause mortality, *N* = 25	Age, baseline DM, CHF, COPD, CT ratio, hs‐CRP, vintage, albumin, and creatinine
Kakiya et al. (2012), Japan^b^	Prospective cohort	Hemodialysis	Diabetic nephropathy, 22% CVD, 29%	494 (63%)	61	NA	125	*DHEA‐S (quartile)* Q1: <443 ng/ml Q2 + Q3 + Q4: ≥443 ng/ml (ref.)	HR (95% CI); F/U = 50 months All‐cause mortality, *N* = 101 (20%) Male all‐cause mortality, *N* = 68 Female all‐cause mortality, *N* = 33	Age, vintage, diabetic nephropathy, BMI, albumin, CRP and pre‐existing CVD, smoking status, HTN, non‐HDL‐C and HDL‐C, ACEI/ARB medication, statin, ESA, use of intravenous iron, and use of VDRA
Relaxin										
Hocher et al. (2004), Germany	Prospective cohort	Hemodialysis	DM, 34% HTN, 90% CHD, 64%	245 (50%)	64	NA	60	*Relaxin (binary)* B1: ≤28.8 pg/ml B2: >28.8 pg/mL (ref.) *Relaxin (per 5 pg/mL)*	RR (95% CI); F/U = 37 months CVD mortality, *N* = 66 (27%) All‐cause mortality, *N* = 107 (44%)	‐

Abbreviations: ACEI, angiotensin converting enzyme inhibitor; ARB, angiotensin II receptor blocker; B, binary; BMI, body mass index; CAD, coronary artery disease; CHD, coronary heart disease; CHF, congestive heart failure; COPD, chronic obstructive peripheral disease; CRP, c‐reactive protein; CT ratio, cardiothoracic ratio; CVD, cardiovascular disease; DHEA‐S, dehydroepiandrosterone sulfate; DM, diabetes mellitus; E, estradiol; eGFR, estimated glomerular filtration rate; ESA, erythrocytosis‐stimulating agent; ESKD, end‐stage kidney disease; FMD, flow‐mediated dilation; FT, free testosterone; F/U, follow‐up; HDL‐C, high‐density lipoprotein cholesterol; HIV, human immunodeficiency virus; HR, hazard ratio; HTN, hypertension; IMT, intima‐media thickness; MAP, mean arterial pressure; MI, myocardial infarction; NA, not applicable; ND, no data; OR, odds ratio; PVD, peripheral vascular disease; PWV, pulse‐wave velocity; Q, quartile; Qi, quintile; RR, relative risk; SHBG, sex hormone‐binding globulin; T, tertile; TRT, testosterone replacement therapy; TT, total testosterone; VDRA, vitamin D receptor activator.

^a^ Data are presented as in the original article (mean or median).

^b^ The study included both male and female patients, but HR was reported only for the male patients.

Limited studies included information on patients receiving hormone replacement therapy. Khurana et al. included participants receiving testosterone replacement therapy who were categorized into the reference group (Khurana et al., 2014). Tanrisev et al. reported that 14 out of 147 patients were on hormone replacement therapy (Tanrisev et al., 2013). Three studies excluded participants who used exogenous hormones (Carrero et al., 2012; Hsu et al., 2012; Kyriazis et al., 2011). Yilmaz et al. reported that no patients were on testosterone or androgen replacement therapy (Yilmaz et al., 2011). Yu et al. were unable to determine which patient received testosterone replacement therapy due to data limitations (Yu et al., 2017).

### 3.3 Quality of included studies

According to the NHLBI Quality Assessment Tool for Observational Cohort and Cross‐Sectional Studies, 12% (*n* = 2) were in excellent quality and 88% (*n* = 15) in good quality (Table S3). In the meta‐analysis, there was a low likelihood of a publication bias of the included studies based on the symmetry observed in funnel plots (Figure S1).

### 3.4 Sex hormones and the risk of CVD events, CVD mortality, and all‐Cause mortality

#### 3.4.1 Testosterone

Four studies examined the association between total testosterone and the risk of CVD events in the CKD population (Table 3). A higher total testosterone level was associated with a higher odds of CVD events in male patients on dialysis [OR 2.51 (1.32–4.76)] (Carrero et al., 2011). In male patients with non‐dialysis stage 1–5 CKD, higher levels of total testosterone and free testosterone were associated with a lower risk of CVD events [HR 0.83 (0.78–0.88) and 0.65 (0.53–0.80), for total testosterone and free testosterone, respectively] (Yilmaz et al., 2011). However, two studies reported no association (Bello et al., 2014; Nakashima et al., 2017).

**TABLE 3:** Testosterone and the risk of CVD events, CVD mortality, and all‐cause mortality

Author, year	Clinical condition, *N* (male%)	Main findings	Adjusted HR, OR, or RR (95% CI)
CVD events			
Bello et al. (2014)	Hemodialysis, 623 (100%)	There was no significant association between TT level and CVD events	Incident CVD events; OR T1: 1.38 (0.60–3.19) T2: 1.61 (0.69–3.74) T3: 1.00
Carrero et al. (2011)	Hemodialysis, 260 (100%)	A higher TT level was associated with lower CVD events	CVD events; OR B1: 2.51 (1.32–4.76) B2: 1.00
Nakashima et al. (2017)	Hemodialysis, 902 (100%)	There was no significant association between TT level and the risk of CVD events	CVD events; HR T1: 1.19 (0.74–1.91) T2: 1.35 (0.86–2.15) T3: 1.00
Yilmaz et al. (2011)	Non‐dialysis CKD (stage 1–5), 239 (100%)	Higher TT and FT levels were associated with a lower risk of CVD events	CVD events; HR TT Cont.: 0.83 (0.78–0.88) FT Cont.: 0.65 (0.53–0.80)
CVD mortality			
Carrero et al. (2009)	Hemodialysis, 126 (100%)	There was no significant association between TT level and the risk of CVD mortality	CVD mortality; HR T1: 2.00 (0.80–4.95) T2 + T3: 1.00
Kyriazis et al. (2011)	Hemodialysis, 111 (100%)	There was no significant association of TT and FT levels with the risk of CVD mortality	CVD mortality; HR TT B1: 2.29 (0.78–6.72) B2: 1.00 T1: 2.48 (0.90–6.85) T2 + T3: 1.00 FT T1: 2.47 (0.92–6.64) T2 + T3: 1.00
Wu et al. (2018)	Hemodialysis, 137 (100%)	A higher TT level was associated with a lower risk of CVD mortality	CVD mortality; HR T1: 6.13 (2.27–16.53) T2 + T3: 1.00
All‐cause mortality			
Bello et al. (2014)	Hemodialysis, 623 (100%)	There was no significant association between TT level and the risk of all‐cause mortality. However, there was a statistically significant trend for a lower all‐cause mortality with a higher TT level (*p* < 0.001)	All‐cause mortality; HR T1: 1.48 (0.82–2.66) T2: 1.32 (0.72–2.42) T3: 1.00
Carrero et al. (2009)	Hemodialysis, 126 (100%)	There was no significant association between TT level and the risk of all‐cause mortality	All‐cause mortality; HR B1: 1.51 (0.86–2.72) B2: 1.00
Carrero et al. (2011)	Hemodialysis, 260 (100%)	A higher TT level was associated with a lower all‐cause mortality	All‐cause mortality; OR B1: 2.00 (1.01–3.97) B2: 1.00
Gungor et al. (2010)	Hemodialysis, 420 (100%)	There was no significant association between TT level and the risk of all‐cause mortality	All‐cause mortality; HR T1: 1.49 (0.86–2.66) T2: 0.76 (0.38–1.54) T3: 1.00 Cont.: 0.96 (0.89–1.02)
Grossmann et al. (2015)	Non‐dialysis stage 3–4 CKD, undergoing dialysis, and kidney transplant recipients, 221 (65%)	A higher TT level was associated with a lower risk of all‐cause mortality in male patients No reports in female patients	All‐cause mortality; HR Cont.: 0.93 (0.88–0.99)
Khurana et al. (2014)	Non‐dialysis stage 3–4 CKD, 2419 (100%)	A higher TT level was associated with a lower risk of all‐cause mortality	All‐cause mortality; HR Qi1: 1.42 (0.995–2.02) Qi2: 1.53 (1.09–2.16) Qi3: 1.22 (0.86–1.73) Qi4: 1.01 (0.70–1.45) Qi5: 1.00 Cont.: 0.70 (0.55–0.89)
Kyriazis et al. (2011)	Hemodialysis, 111 (100%)	Higher levels of TT and FT were associated with a lower risk of all‐cause mortality	All‐cause mortality; HR TT B1: 2.81 (1.23–6.38) B2: 1.00 T1: 4.04 (1.86–8.76) T2 + T3: 1.00 FT T1: 2.62 (1.27–5.44) T2 + T3: 1.00
Nakashima et al. (2017)	Hemodialysis, 902 (100%)	A higher TT level was associated with a lower risk of all‐cause mortality	All‐cause mortality; HR T1: 2.26 (1.21–4.23) T2: 1.69 (0.87–3.28) T3: 1.00
Wu et al. (2018)	Hemodialysis, 137 (100%)	A higher TT level was associated with a lower risk of all‐cause mortality	All‐cause mortality; HR T1: 3.39 (1.67–6.86) T2 + T3: 1.00
Yu et al. (2017)	Hemodialysis and peritoneal dialysis, 624 (100%)	A higher TT level was associated with a lower risk of all‐cause mortality	All‐cause mortality; HR Q1: 2.32 (1.33–4.06) Q2: 1.80 (0.99–3.28) Q3: 1.00 Q4: 0.68 (0.32–1.42)

Abbreviations: B, binary; CKD, chronic kidney disease; Cont., continuous; CVD, cardiovascular disease; DHEA‐S, Dehydroepiandrosterone sulfate; FT, free testosterone; HR, hazard ratio; OR, odds ratio; Q, quartile; Qi, quintile; RR, relative risk; T, tertile; TT, total testosterone.

**FIGURE 2:**
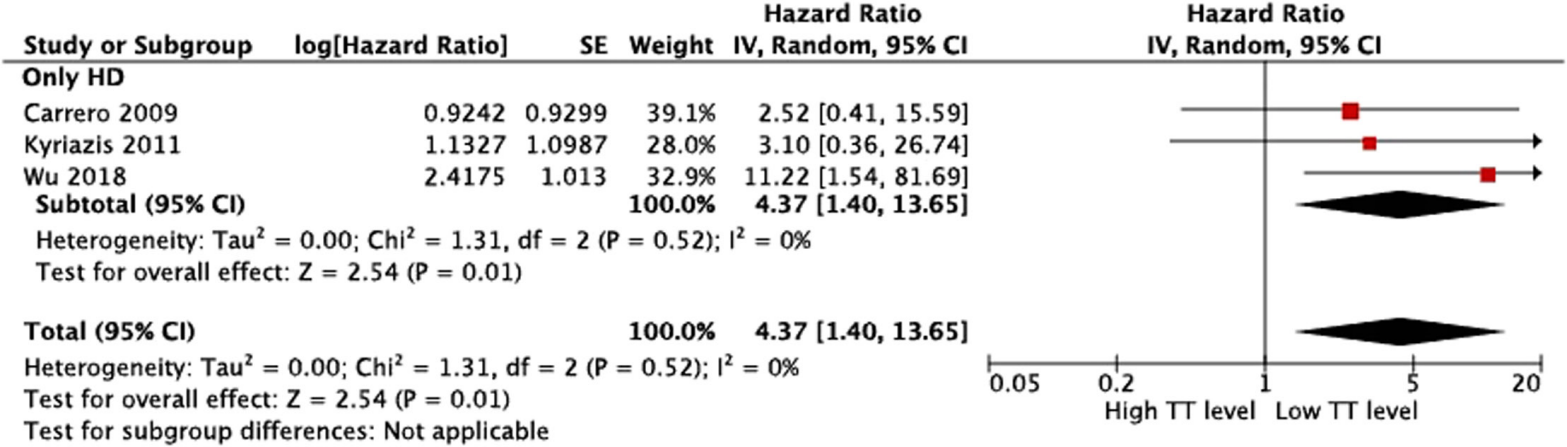
Random‐effects pooled hazard ratio of CVD death comparing highest versus lowest tertile of TT level in patients on hemodialysis. CI, confidence interval; CKD, chronic kidney disease; CVD, cardiovascular disease; HD, hemodialysis; SE, standard error; TT, total testosterone.

Three studies examined the association between total testosterone level and the risk of CVD mortality in male patients on hemodialysis (Table 3). Wu et al. demonstrated a higher total testosterone level was associated with a lower CVD mortality risk in male patients on hemodialysis [HR 6.13 (2.27–16.53)] (Wu et al., 2018). However, two studies reported no association (Carrero et al., 2009; Kyriazis et al., 2011). In the meta‐analysis (Carrero et al., 2009; Kyriazis et al., 2011; Wu et al., 2018), a lower total testosterone level was associated with a higher risk of CVD mortality [pooled HR 4.37 (1.40–13.65)], with low heterogeneity among studies (Figure 2).

**FIGURE 3:**
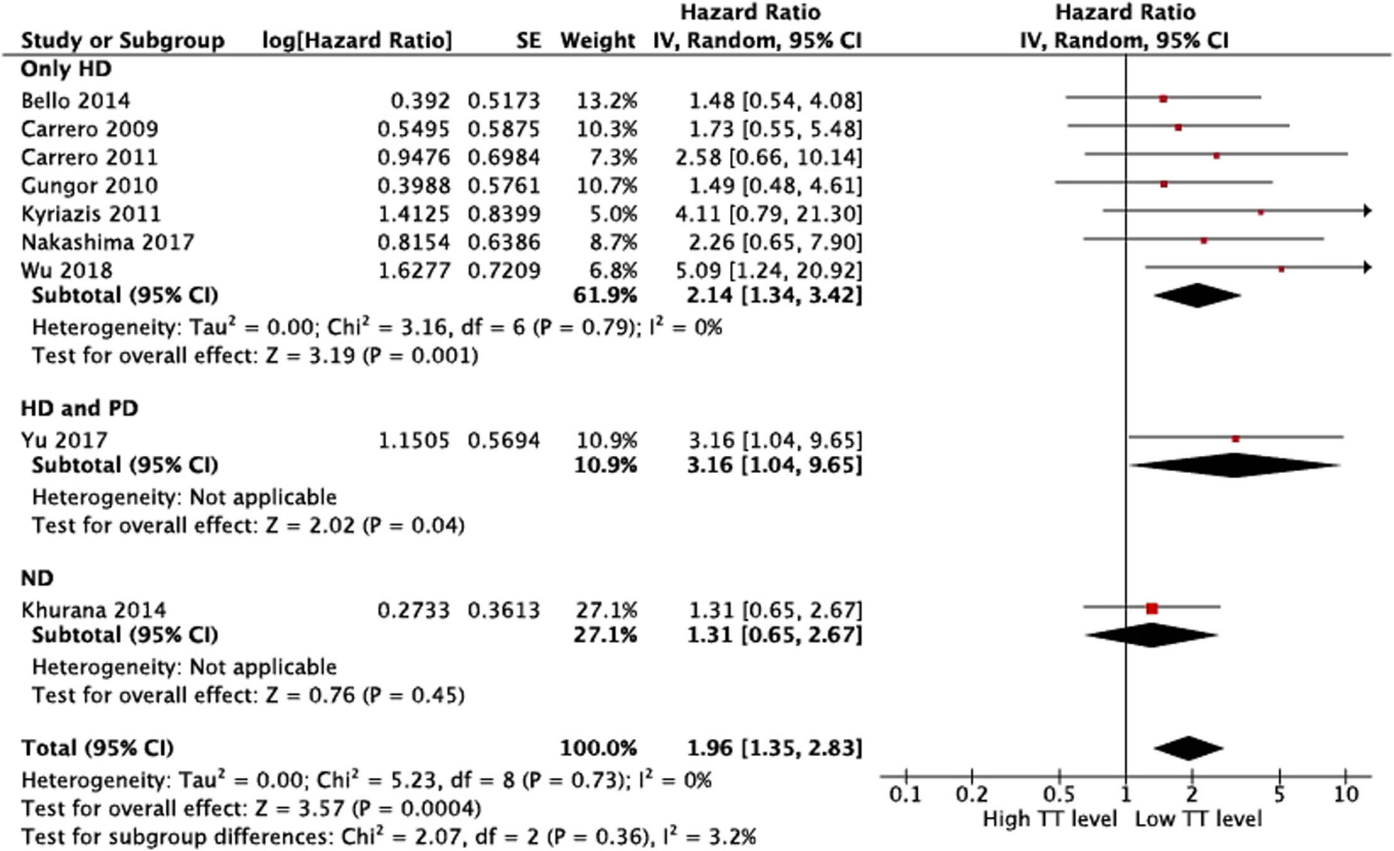
Random‐effects pooled hazard ratio of all‐cause death comparing highest versus lowest tertile of TT level in patients with dialysis and non‐dialysis CKD. CI, confidence interval; CKD, chronic kidney disease; HD, hemodialysis; PD, peritoneal dialysis; SE, standard error; TT, total testosterone.

Ten studies evaluated the association between total testosterone level and CVD mortality risk in males with CKD (Table 3). A higher total testosterone level was associated with a lower risk of all‐cause mortality in male patients with non‐dialysis CKD stage 3–4 [HR 0.70 (0.55–0.89)] (Khurana et al., 2014) and those with non‐dialysis CKD and those on dialysis and kidney transplant [HR 0.93 (0.88–0.99)] (Grossmann et al., 2015). In five studies that included male patients on dialysis, a higher total testosterone level was associated with a lower all‐cause mortality risk (Carrero et al., 2011; Kyriazis et al., 2011; Nakashima et al., 2017; Wu et al., 2018; Yu et al., 2017). However, three studies reported no association (Bello et al., 2014; Carrero et al., 2009; Gungor et al., 2010). In the meta‐analysis (Bello et al., 2014; Carrero et al., 2009, 2011; Gungor et al., 2010; Khurana et al., 2014; Kyriazis et al., 2011; Nakashima et al., 2017; Wu et al., 2018; Yu et al., 2017), a lower total testosterone level was associated with a higher risk of all‐cause mortality [pooled HR 1.96 (1.35–2.83)], with low heterogeneity among studies (Figure 3). Similar results were observed in the meta‐analysis of studies including male patients on hemodialysis only [pooled HR 2.14 (1.34–3.42)].

#### 3.4.2 Estradiol

Two studies examined the association between circulating estradiol levels and the risk of CVD and all‐cause mortality in female patients with CKD (Table 4). A U‐shaped association between estradiol and the risk of CVD mortality [HR 5.13 (1.29–20.3) and 4.21 (1.17–15.1)] and all‐cause mortality [HR 4.49 (1.59–12.6) and 4.32 (1.59–11.7), for lowest and highest tertiles of estradiol, respectively] was reported in females on hemodialysis (Tanrisev et al., 2013). Ramesh et al. demonstrated a higher estradiol level was associated with a higher risk of all‐cause mortality, but not CVD mortality, in females on hemodialysis [HR 1.86 (1.14–3.01)] (Ramesh et al., 2020).

**TABLE 4:** Estradiol and the risk of CVD events, CVD mortality, and all‐cause mortality

Author, year	Clinical condition, N (male%)	Main findings	Adjusted HR, OR, or RR (95% CI)
CVD mortality			
Tanrisev et al. (2013)	Hemodialysis, 147 (0%)	There was a U‐shaped association between E level and the risk of CVD mortality	CVD mortality; HR T1: 5.13 (1.29–20.3) T2: 1.00 T3: 4.21 (1.17–15.1)
Ramesh et al. (2020)	Hemodialysis, 476 (0%)	There was no significant association between E and CVD mortality	CVD mortality; HR Qi1: 1.00 Qi2: 1.30 (0.60–2.83) Qi3: 1.07 (0.47–2.44) Qi4: 1.48 (0.67–3.28) Qi5: 2.02 (0.90–4.54)
All‐cause mortality			
Tanrisev et al. (2013)	Hemodialysis, 147 (0%)	There was a U‐shaped association between E level and the risk of all‐cause mortality	All‐cause mortality; HR T1: 4.49 (1.59–12.6) T2: 1.00 T3: 4.32 (1.59–11.7)
Ramesh et al. (2020)	Hemodialysis, 476 (0%)	A higher E level was associated with a higher risk of all‐cause mortality	All‐cause mortality; HR Qi1: 1.00 Qi2: 1.31 (0.84–2.03) Qi3: 1.35 (0.86–2.12) Qi4: 2.16 (1.41–3.31) Qi5: 1.86 (1.14–3.01)

Abbreviations: CVD, cardiovascular disease; E, estradiol; HR, hazard ratio; OR, odds ratio; Qi, quintile; RR, relative risk; T, tertile.

#### 3.4.3 Prolactin

One study examined the association of prolactin levels with the risk of CVD events, CVD mortality, and all‐cause mortality in patients with non‐dialysis CKD and those on hemodialysis (Table 5). In male and female patients with non‐dialysis CKD, a higher prolactin level was associated with a higher risk of CVD events [HR 1.19 (1.08–1.32)] (Carrero et al., 2012). In males and females on hemodialysis, a higher prolactin level was associated with a higher risk of CVD and all‐cause mortality [HR 1.13 (1.05–1.21) and 1.10 (1.04–1.17), respectively] (Carrero et al., 2012).

**TABLE 5:** Prolactin and the risk of CVD events, CVD mortality, and all‐cause mortality

Author, year	Clinical condition, N (male%)	Main findings	Adjusted HR, OR, or RR (95% CI)
CVD events			
Carrero et al. (2012)	Non‐dialysis CKD, 457 (50%)	A higher P level was associated with a higher risk of CVD events	CVD events; HR Cont.: 1.19 (1.08–1.32)
CVD mortality			
Carrero et al. (2012)	Hemodialysis, 173 (64%)	A higher P level was associated with a higher risk of CVD mortality	CVD mortality; HR Cont.: 1.13 (1.05–1.21)
All‐cause mortality			
Carrero et al. (2012)	Hemodialysis, 173 (64%)	A higher P level was associated with a higher risk of all‐cause mortality	All‐cause mortality; HR Cont.: 1.10 (1.04–1.17)

Abbreviations: B, binary; CKD, chronic kidney disease; Cont., continuous; CVD, cardiovascular disease; HR, hazard ratio; OR, odds ratio; P, prolactin; RR, relative risk.

#### 3.4.4 Dehydroepiandrosterone sulfate (DHEA‐S)

Two studies evaluated the association between DHEA‐S level and the risk of CVD and all‐cause mortality in male patients on hemodialysis (Table 6). Hus et al. demonstrated a lower DHEA‐S level was associated with a higher risk of CVD and all‐cause mortality [HR 3.81 (0.91–15.93) and 2.93 (1.09–7.89), respectively] (Hsu et al., 2012). Kakiya et al. reported a lower DHEA‐S level was associated with a greater risk of all‐cause mortality [*n* = 31, HR 2.47 (1.46–4.19)] (Kakiya et al., 2012).

**TABLE 6:** Dehydroepiandrosterone sulfate (DHEA‐S) and the risk of CVD mortality and all‐cause mortality

Author, year	Clinical condition, N (male%)	Main findings	Adjusted HR, OR, or RR (95% CI)
CVD mortality			
Hsu et al. (2012)	Hemodialysis, 200 (47%)	There was no significant association between DHEA‐S level and the risk of CVD mortality in male patients No reports in female patients	CVD mortality; HR B1: 3.81 (0.91–15.93) B2: 1.00 Cont.: 1.00 (0.999–1.001)
All‐cause mortality			
Hsu et al. (2012)	Hemodialysis, 94 (100%)	A lower DHEA‐S level was associated with a higher risk of all‐cause mortality	All‐cause mortality; HR B1: 2.93 (1.09–7.89) B2: 1.00 Cont.: 1.00 (0.999–1.001)
Kakiya et al. (2012)	Hemodialysis, 494 (63%)	A lower DHEA‐S level was associated with a higher risk of all‐cause mortality in male patients No reports in female patients	All‐cause mortality; HR Q1: 2.47 (1.46–4.19) Q2 + Q3 + Q4: 1.00

Abbreviations: B, binary; Cont., continuous; CVD, cardiovascular disease; DHEA‐S, Dehydroepiandrosterone sulfate; HR, hazard ratio; OR, odds ratio; Q, quartile; RR, relative risk.

#### 3.4.5 Relaxin

One study reported the association between relaxin and the risk of CVD and all‐cause mortality in patients on hemodialysis (Table 7). A higher relaxin level was associated with a greater risk of CVD and all‐cause mortality in male patients with end‐stage kidney disease [RR 2.95 (1.20–7.21) and 2.63 (1.34–5.12), respectively], but not in female patients (Hocher et al., 2004).

**TABLE 7:** Relaxin the risk of CVD mortality and all‐cause mortality

Author, year	Clinical condition, N (male%)	Main findings	Adjusted HR, OR, or RR (95% CI)^a^
CVD mortality			
Hocher et al. (2004)	Hemodialysis, 245 (50%)	A higher relaxin level was associated with a higher risk of CVD mortality in male patients, but not in female patients	CVD mortality; RR Male B1: 1.00 B2: 2.95 (1.20–7.21) Cont.: ND Female B1: 1.00 B2: 0.64 (0.26–1.56) Cont.: ND
All‐cause mortality			
Hocher et al. (2004)	Hemodialysis, 245 (50%)	A higher relaxin level was associated with a higher risk of all‐cause mortality in male patients, but not in female patients	All‐cause mortality; RR Male B1: 1.00 B2: 2.63 (1.34–5.12) Cont.: 1.08 (1.02–1.11) Female B1: 1.00 B2: 0.67 (0.33–1.35) Cont.: 1.03 (0.96–1.10)

Abbreviations: B, binary; Cont., continuous; CVD, cardiovascular disease; HR, hazard ratio; ND, no data; OR, odds ratio; RR, relative risk.

a Adjusted HR, OR, or RR (95% CI): Values are HR, unless noted as OR or RR.

## 4 DISCUSSION

This systematic review and meta‐analysis evaluated the findings of the association between circulating sex hormone levels and the risk of CVD events, CVD mortality, and all‐cause mortality in male and female patients with non‐dialysis CKD and those on dialysis. The majority of studies examined circulating total testosterone levels (11 out of 17 studies) and included only male patients with CKD (12 out of 17 studies). In the meta‐analysis, we demonstrated that a lower total testosterone level was associated with a higher risk of CVD and all‐cause mortality. The results from the systematic review and meta‐analysis suggest that a higher circulating total testosterone level is associated with a higher risk of all‐cause mortality in male patients with CKD. However, additional large‐scale observational studies are needed to better determine the association of other circulating sex hormones, including free testosterone, estradiol, prolactin, DHEA‐S, and relaxin, with the risk of cardiovascular outcomes and mortality in the CKD population.

Testosterone has a variety of effects on cardiovascular physiology and pathophysiology (Kaur & Werstuck, 2021). Previous studies demonstrate the vasodilatory effect of testosterone through the downregulation of L‐type voltage‐gated calcium channels (Jones et al., 2002) and the upregulation of calcium‐activated potassium channels (Cairrão et al., 2008). Moreover, a lower testosterone level is associated with a longer heart rate‐correct QT interval (testosterone replacement therapy results in interval shortening) (Charbit et al., 2009), which could elevate the risk for incident ventricular arrhythmia and subsequent sudden cardiac death (Nielsen et al., 2013). Large observational studies report an inverse association of testosterone levels with the risk of CVD in community‐dwelling older males in Sweden (Ohlsson et al., 2011) (*n* = 2416; mean age 75 years; median follow‐up 5 years) and the risk of ischemic stroke in the general male population in Denmark (*n* = 4615; median age 58 years; median follow‐up 20 years) (Holmegard et al., 2016). In the current systematic review and meta‐analysis, we demonstrated that a higher total testosterone level was associated with a lower risk of cardiovascular outcomes and all‐cause mortality in male patients with CKD, suggesting the inverse association in the general male population may translate to the male CKD population despite the decline in total testosterone concentrations with aging (Golan et al., 2015) as well as induced by CKD (Carrero et al., 2011; Yilmaz et al., 2011). Of note, however, the “free hormone hypothesis” proposes only the unbound or free fraction of hormones is able to enter cells and exert biological effects in target tissues, implicating only free testosterone as biologically active (Goldman et al., 2017). In addition, although total testosterone and free testosterone are highly correlated, this correlation may diverge in individuals with altered hormone binding protein concentrations [sex hormone‐binding globulin (SHBG; binds to testosterone with high affinity) and human serum albumin (binds testosterone with lower affinity than SHBG does)], such as patients with CKD (Goldman et al., 2017). We observed only a few studies (2 out of 11 studies) that measured free testosterone level and evaluated its association with the risk of cardiovascular outcomes and mortality. Therefore, more studies are warranted to examine the role of biologically active free testosterone on the risk of CVD and mortality in the CKD population.

A recent study demonstrated differences in circulating testosterone levels according to dialysis modality (hemodialysis vs. peritoneal dialysis) in male patients with CKD (Cigarrán et al., 2017). The level of total testosterone was significantly lower in patients on hemodialysis as compared to those on peritoneal dialysis, suggesting dialysis modality may impact the sex hormone concentrations. Moreover, this study reported this differences in testosterone levels between the dialysis modalities remained significant after adjusting for factors associated with reduced testosterone levels (i.e. age, diabetes, and vintage), and suggested dialysis modality may differently condition testosterone removal through the dialysate or the effluent (Cigarrán et al., 2017). However, further studies are needed to test this hypothesis. In the current meta‐analysis, we were unable to perform a sub‐group analysis for CVD outcomes by dialysis modality due to limited data. Future studies should compare sex hormone concentrations, but not limited to testosterone, in patients on different dialysis modalities (hemodialysis, peritoneal dialysis, and hemodiafiltration), and correlate them with the risk of CVD outcomes and mortality. It might be also interesting to investigate the effect of different types of kidney replacement therapies (transplant vs. dialysis) on sex hormone concentrations and their association with CVD and mortality risk.

In the general female population, large‐scale observational studies report an inverse association between circulating estrogen levels and cardiovascular risk. For example, a lower circulating estrogen level was associated with a higher CVD risk in population‐based cohort studies in the Netherlands (*n* = 9450; mean age 57 years; mean follow‐up 21 years) (De Kleijn et al., 2002) and Denmark (*n* = 4716; median age 59 years; follow‐up ≥30 years) (Benn et al., 2015). In the Multi‐Ethnic Study of Atherosclerosis (MESA) study that included post‐menopausal females (*n* = 2834; mean age 65 years; mean 12 years follow‐up), greater testosterone to estradiol ratio was associated with a higher risk of CVD (Zhao et al., 2018). Estrogen promotes vasodilation by increasing the circulating nitric oxide concentrations, which may enhance vascular function and lower cardiovascular risk (Miller & Duckles, 2008). In the current review, we found only two studies (with conflicting results) that examined the association between circulating estradiol and the risk of CVD and all‐cause mortality in postmenopausal female patients on hemodialysis (Ramesh et al., 2020; Tanrisev et al., 2013). One study reported an association between estradiol levels and all‐cause mortality (Ramesh et al., 2020), while the other study reported a U‐shaped association of estradiol levels with CVD and all‐cause mortality (Tanrisev et al., 2013). The possible discrepancies between these studies could be explained by the small sample size (*n* = 476 vs. *n* = 147, respectively for Ramesh et al. and Tanrisev et al.) and different dialysis vintage (initiating dialysis vs. median 35 months, for Ramesh et al. and Tanrisev et al. respectively). Additional large observational studies are needed to examine the association of estradiol and outcomes in female patients with CKD, including the inclusion of younger patients (pre‐ and peri‐menopausal ages) and those with earlier stages of CKD (stage 2–4 CKD).

The effect of hormone (testosterone or estrogen) replacement therapy (HRT) on the general population has been extensively investigated and is controversial due to studies reporting its adverse effect on cardiovascular risk. Notably in females, while observational studies uniformly showed a beneficial effect of HRT, randomized controlled trials suggested a harmful effect, particularly in females who were many years apart from menopause (Humphrey et al., 2002; Magliano et al., 2006). However, recently, the “timing hypothesis” proposes that HRT started in the premenopausal or early postmenopausal period is cardioprotective, whereas HRT started later postmenopausal period increases the risk of CVD (Giordano et al., 2015). Detailed reviews on whom, when, and how the HRT should be offered and its associated risk for CVD have been discussed elsewhere (Giordano et al., 2015; Tsametis & Isidori, 2018). In the CKD population, a few studies report the beneficial effect of HRT on reduced risk of CKD progression and all‐cause mortality in male patients with end‐stage kidney disease (Sharma et al., 2020) and reduced urinary albumin‐creatinine ratio cross‐sectionally in post‐menopausal female patients with CKD who used HRT vs. non‐users (Schopick et al., 2009). However, to the best of our knowledge, no studies to date have investigated the impact of HRT on cardiovascular outcomes in the male and female CKD population, which implicates a strong need for future observational and interventional studies to identify whether correction of testosterone or estrogen deficiency reduces cardiovascular outcomes and mortality risk and an optimal target range of testosterone and estradiol levels in patients with CKD.

We also found several studies examining the association of other sex hormones, including prolactin, DHEA‐S, and relaxin, with the risk of cardiovascular outcomes and mortality in male and female patients with CKD. Prolactin is a hormone that can regulate vessel formation and cardiac remodeling (Corbacho & Clapp, 2002), which leads to defective cardiac angiogenesis, heart failure and subsequent mortality (Oka et al., 2014). In the general male and female population, endogenous prolactin levels are associated with a higher risk for CVD and all‐cause mortality (Haring et al., 2014). In the CKD population, circulating levels of prolactin are increased, which may result from reduced renal clearance (Yavuz et al., 2005) and reduced sensitivity to dopaminergic inhibition and thus upregulated production (Mckenna & Woolf, 1985). We identified one study reporting a positive association between prolactin level and the risk for incident CVD, CVD mortality, and all‐cause mortality in male and female patients with non‐dialysis CKD (Carrero et al., 2012). DHEA‐S, produced predominantly by the adrenal glands (Neunzig & Bernhardt, 2014), is the most abundant endogenous steroid hormone both in males and females (Shufelt et al., 2010). Although the role of DHEA‐S in CVD is still not clear, emerging studies have reported an inverse association between DHEA‐S and CVD risk in the general population (Jia et al., 2020; Jiménez et al., 2019). A lower concentration of circulating DHEA‐S is observed in male patients on dialysis (Inaudi et al., 1983; Mastrogiacomo et al., 1988; Vasdev et al., 1987). In the current review, we identified two studies that examined DHEA‐S (Hsu et al., 2012; Kakiya et al., 2012). While DHEA‐S was not associated with CVD mortality, lower DHEA‐S was associated with a higher risk of all‐cause mortality in male and female patients on hemodialysis (Hsu et al., 2012; Kakiya et al., 2012). Relaxin, a peptide hormone and a member of the insulin family (Bathgate et al., 2013), plays a cardioprotective role against myocardial injury, vasoconstriction, oxidative stress, fibrosis, and inflammation (Du et al., 2010). We identified one study reporting higher relaxin is associated with a greater risk of CVD and all‐cause mortality in male patients on hemodialysis, but not in female patients (Hocher et al., 2004). It is possible there may be a compensatory increase in circulating relaxin concentrations in response to cardiac dysfunction, but the mechanisms underlying this association are unknown. Given the differing roles of prolactin, DHEA‐S, and relaxin on the vasculature, further studies are needed to examine this association in the CKD population.

The current systematic review and meta‐analysis assessed the association of circulating sex hormone concentrations with the risk of cardiovascular outcomes and mortality in the CKD population. A recent systematic review was published while our systematic review was under review. In this recent systematic review, a pooled analysis of studies that examined the association of testosterone and DHEA‐S with CVD events and mortality in patients with CKD, and 9 studies (testosterone *n* = 7; DHEA‐S *n* = 2) were identified (van der Burgh et al., 2022). However, we identified a total of 13 studies (testosterone *n* = 11; DHEA‐S *n* = 2), suggesting we implemented a comprehensive and complete search strategy which is more robust and extensive as compared to the previously reported systematic review. Moreover, the current review includes other sex hormones, such as estradiol, prolactin, and relaxin, which has not been previously examined. Furthermore, the current meta‐analysis presented a low heterogeneity among studies. There are also several limitations. All studies included in this review are prospective cohort studies; thus, the results are observational rather than causal due to the nature of the study design. The included studies adjusted for important covariates, but the results may be subject to residual confounding, via factors either unmeasured or unknown at this time. For example, when adjusted for SHBG, the association between total testosterone with the risk of metabolic syndrome was no longer significant in males who participated in the Framingham Heart Study (Bhasin et al., 2011), suggesting a confounding effect of SHBG. We found only two studies that included SHBG in the adjusted model. Moreover, the timing of blood sample collection might have had a confounding effect on the association observed in the included studies, since it may not have been possible to collect a fasting blood sample from patients on dialysis. Most studies included in this review examined circulating testosterone levels in male patients with CKD, those older in age (mean/median age range from 52 to 72 years), and those with advanced CKD requiring dialysis. Thus, further studies are warranted to examine the association of circulating sex hormone concentrations with cardiovascular risk in females with mild‐to‐moderate CKD and younger patients. Additionally, the concentration of endogenous sex hormones in females changes across the menopause transition. Thus, future studies should focus on the association of changes in circulating sex hormone levels induced by menopause, CKD, or both with the risk of cardiovascular outcomes in pre‐, peri‐, and post‐menopausal females with CKD.

In summary, through a systematic review and meta‐analysis, we observed an inverse association between circulating total testosterone levels and the risk of cardiovascular and all‐cause mortality in male patients with CKD. However, further research is needed to examine the association of sex hormones in female patients with CKD, including differences across the menopause transition, as well as in the role of sex hormones in cardiovascular risk in earlier stages of CKD.

## AUTHOR CONTRIBUTIONS

ESO, AJJ, and KLN designed the study. ESO and CNS conducted the study, collected data, and verified the data. EO, CNS, and ZY participated in data analysis. ESO, AJJ, and KLN participated in data interpretation and wrote the paper. All authors contributed to the article and approved the final version of submitted manuscript.

## FUNDING INFORMATION

The authors declare that they have no relevant financial interest.

## Supporting information


Appendix S1.
